# Evaluation of Recent Advanced Soft Computing Techniques for Gully Erosion Susceptibility Mapping: A Comparative Study

**DOI:** 10.3390/s20020335

**Published:** 2020-01-07

**Authors:** Alireza Arabameri, Thomas Blaschke, Biswajeet Pradhan, Hamid Reza Pourghasemi, John P. Tiefenbacher, Dieu Tien Bui

**Affiliations:** 1 Department of Geomorphology, Tarbiat Modares University, Tehran 36581-17994, Iran; alireza.ameri91@yahoo.com; 2 Department of Geoinformatics—Z_GIS, University of Salzburg, 5020 Salzburg, Austria; thomas.blaschke@sbg.ac.at; 3 Centre for Advanced Modelling and Geospatial Information Systems (CAMGIS), Faculty of Engineering and IT, University of Technology Sydney, Ultimo, NSW 2007, Australia; biswajeet.pradhan@uts.edu.au; 4 Department of Energy and Mineral Resources Engineering, Choongmu-gwan, Sejong University, 209 Neungdong-ro, Gwangjin-gu, Seoul 05006, Korea; 5 Department of Natural Resources and Environmental Engineering, College of Agriculture, Shiraz University, Shiraz 71441-65186, Iran; 6 Department of Geography, Texas State University, San Marcos, TX 78666, USA; tief@txstate.edu; 7 Institute of Research and Development, Duy Tan University, Da Nang 550000, Vietnam

**Keywords:** gully erosion, GIS, soft computing, hybrid model, ensemble, Iran

## Abstract

Gully erosion is a problem; therefore, it must be predicted using highly accurate predictive models to avoid losses caused by gully development and to guarantee sustainable development. This research investigates the predictive performance of seven multiple-criteria decision-making (MCDM), statistical, and machine learning (ML)-based models and their ensembles for gully erosion susceptibility mapping (GESM). A case study of the Dasjard River watershed, Iran uses a database of 306 gully head cuts and 15 conditioning factors. The database was divided 70:30 to train and verify the models. Their performance was assessed with the area under prediction rate curve (AUPRC), the area under success rate curve (AUSRC), accuracy, and kappa. Results show that slope is key to gully formation. The maximum entropy (ME) ML model has the best performance (AUSRC = 0.947, AUPRC = 0.948, accuracy = 0.849 and kappa = 0.699). The second best is the random forest (RF) model (AUSRC = 0.965, AUPRC = 0.932, accuracy = 0.812 and kappa = 0.624). By contrast, the TOPSIS (Technique for Order Preference by Similarity to Ideal Solution) model was the least effective (AUSRC = 0.871, AUPRC = 0.867, accuracy = 0.758 and kappa = 0.516). RF increased the performance of statistical index (SI) and frequency ratio (FR) statistical models. Furthermore, the combination of a generalized linear model (GLM), and functional data analysis (FDA) improved their performances. The results demonstrate that a combination of geographic information systems (GIS) with remote sensing (RS)-based ML models can successfully map gully erosion susceptibility, particularly in low-income and developing regions. This method can aid the analyses and decisions of natural resources managers and local planners to reduce damages by focusing attention and resources on areas prone to the worst and most damaging gully erosion.

## 1. Introduction

By relying on the principles of systemic view, geomorphology can help understand the mechanisms governing the natural environment. This knowledge enables humans to act in such a way that their activities will not damage the natural environment and instead complement natural processes. Nature-based solutions have been highlighted as a superior approach to land management based on engineered structures, which are still preferred by many landowners and managers [1]. To work with the landscape, geomorphologists, using their knowledge of natural morpho-dynamic factors, can predict environmental responses to prospective remedies to ensure that ecosystem services will be preserved or even restored. Before designing solutions to stop degradation and enable restoration, it is wise to understand the current state of the land.

Given the destructive effects of gully erosion (GE), solutions for managing this phenomenon to achieve sustainable development are essential [2]. Gully erosion-susceptibility mapping (GESM) is one basic method [3] to understand the mechanisms behind gully erosion. To predict the patterns of GE, a gully-erosion inventory and methods to identify and measure pertinent gully-erosion conditioning factors (GECFs) are needed [4]. Technically speaking, drainage networks, soil characteristics, rainfall, land use, topography, and lithology have been identified as the relevant GEFs controlling gully erosion and development [5].

A geographic information system (GIS), remote sensing (RS), and statistical data analyses are indispensable tools for examination of multidimensional outcomes like GE. Several factors are potential influences [3]. A variety of GIS-based approaches for GESM have been proposed and they can be classified into three types: multicriteria decision-making (MCDM), statistical modeling, and machine learning (ML) models. MCDM models are based on the knowledge of decision makers to identify, select, and weight conditioning factors [6,7,8,9,10]. These factors are combined to develop a GE model. Although recent developments in mathematical science, computational science, and computer technology have yielded more than 20 new MCDM models, the ranking of factors remains subjective. Statistical models provide a general advantage of working with diverse types of independent variables, like continues, binary, and categorical data [5]. The most successful models may be: information value (IV) [5], conditional probability [11], frequency ratio (FR) [12], evidential belief function [3], index of entropy (IoE) [13], certainty factor [14], weights of evidence (WOE) [15], and logistic regression [16]. The performance of statistical models is low, however. ML has proven to be efficient for GE modeling due to its ability to handle small training sets and factors with complex relationships. The most successful ML models for GE consist of multivariate adaptive regression spline [17], maximum entropy (ME) [18], boosted regression tree [19], artificial neural network (ANN) [20], random forest (RF) [21], linear discriminant analysis [22], bagging best-first decision tree [23], support vector machine [24], classification and regression trees [20], and flexible discriminant analysis [14], generalized linear model (GLM) [25], functional data analysis (FDA) [26], and the technique for order preference by similarity to the ideal solution (TOPSIS) [27].

Many ML models have been used by researchers, each has its disadvantages and advantages [22]. The selection of a suitable method is critical and requires careful consideration [24]. A comprehensive comparative assessment of MCDM, statistical, and ML models for GESM for use in arid and semi-arid regions of the world has not been conducted. This research attempts to begin to fill this gap by comparing seven models—MCDM-based TOPSIS, the statistically based statistical index (SI) and FR, and the ML-based RF, ME, GLM, and FDA—for GESM. SI is a simple and quantitatively suitable model that has been applied to landslide-susceptibility mapping [28,29], but this is the first time it has been used for GESM.

Ensemble models, combinations of two or more statistical and ML techniques, have been proven to work for GESM [3,11]. Theoretically, ensemble models inherit the virtues and eliminate the shortcomings of individual techniques to form more robust models [30,31]. They ensure diversity to guarantee high prediction performance of their models. Ensemble models for gully-erosion modeling can be classified into simple integration models, homogeneous frameworks, and heterogeneous frameworks [32]. Simple integration is a simple assemblage of individual methods. A homogenous framework (i.e., boosting, bagging, rotation forest) creates subsets from the original training set, uses a ML algorithm to generate a classifier for each subset, and groups the classifiers into an ensemble model. This procedure is also used for heterogenous frameworks. The difference is that different ML algorithms are used for each of the subsets to create classifiers. Errors are dramatically reduced by combining independent learners into ensemble models [30]. This study also combines models to achieve a better collective performance to map gully-erosion susceptibility.

The study was conducted in the Dasjard River watershed, Iran. This watershed is an arid region [33] that has experienced severe GE in recent years. The aims of this study are to explore the capabilities of several individual and ensemble approaches for GESM, to evaluate the influences of GECFs on GE, and to validate the modeled susceptibility maps using several criteria. Comparison of MCDM, statistical, and ML approaches and their ensembles with a RS dataset to evaluate several GEFs for GESM is novel to this study.

## 2. Description of the Study Area

**Figure 1:**
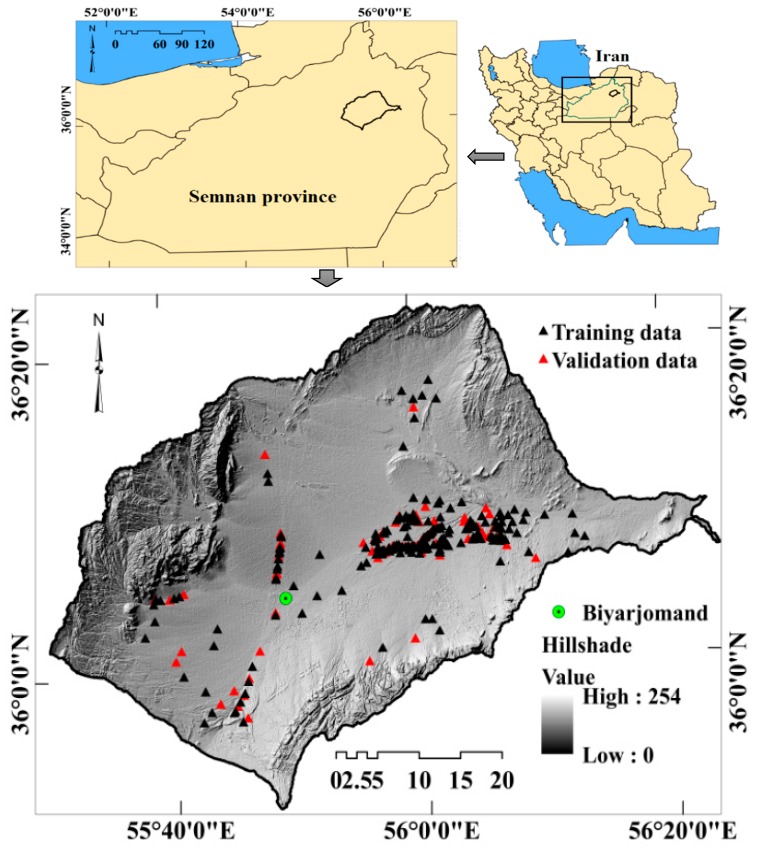
Location of study area in Semnan province and Iran, and location of training and validation gullies in the study area.

The Dasjard River watershed is found between 35°51′24″ and 36°22′32″ N and between 55°29′54″ and 56°23′14″ E (Figure 1), covering 2820.29 km^2^. Elevation ranges from 793 to 2418 m above sea level (m.a.s.l.). The steepest slope in the watershed is 73° and the mean is 3.8°. The central portion of the watershed is generally flat. In fact, 36.5% of the study area is a relatively flat plain. Precipitation ranges from 47.34 mm to 230.43 mm annually across the region, but the average is 154.3 mm. More than 75% of the precipitation occurs in December and January [33]. The mean annual temperature is 17.8 °C and the range is from 43 °C to −6 °C [33]. The climates across the watershed are arid and semiarid [33].

Reading from the 1:100,000 scale Toroud sheet from the Geological Survey Department of Iran [34], the region is covered by Quaternary lithotypes. Clayey material, well-sorted sand dunes, salt concretions, mixed terrace deposits, and swamp or marsh deposits are the most important units [34]. The geological structure of the study area is crossed by an important E-to-W Quaternary strike fault (the Toroud fault) that is responsible for uplift of a metamorphic basement in the northern part of the study area. Morphologically, steep slopes (average > 30°) dominate the northern third of the area. High local relief is a product of heavy dissection of an uplifted surface. These slopes display the rectilinear-convex profile of V-shaped valleys. The central third of the watershed has gentle slopes where Quaternary deposits are found as outcrops. Denudation from mass wasting and water erosion has significantly affected hill slopes. The profiles are well articulated with concave or convex shapes and are incised by concave valleys. The landscape of the middle of the watershed is characterized by terraces, deeply dissected by V-shaped or concave valleys. More recent fluvial terraces and alluvial fans are also commonplace. Several soil types are found in the Dasjard River watershed. The soils are poorly developed (Aridisols and Entisols) [35] and frequently appear truncated or strongly degraded at the surface by water erosion.

The gully features in the study region range from 0.79 m to 365 m in length, and are at least 0.65 m deep, but can be as much as 7.2 m deep. The widths of gullies range from 0.76 m to 19.4 m. These formations reflect the primary mechanism of landscape degradation in the area, particularly soil erosion. Water erosion occurs relatively slowly over long periods of time, but even relatively small rainfall events can yield significant gully incision and retrogradation. Field monitoring of gully head cuts in the area has distinguished lateral erosion, primarily caused by instability of the perimeter edges of the incision, which leads to gravitational collapse ranging from micro- to meso-scale impacts. The main gullies have V- and U-shaped cross-sections that retrograde into steep, unstable scarps. The river valley contains gullies that formed on both river terraces and slopes. Gullying degrades agricultural land, roadways, and irrigation canals, threatening settlements and local economies.

Piping is an important process related to gully erosion. Piping dissolves soluble soil materials and disaggregates loose soil. Like sinkholes, pipes undercut structures and create tunnels. These features are most often caused by infiltration into susceptible materials and subsequent shallow groundwater flows. The Biarjamand watershed has extensive deposits of fine-grained (silt and clay) soils and in soils with soluble (salt, gypsum, and carbonate) mineral fractions. The former, due to their clay content, expand and contract as they moisten and desiccate. During dry seasons, the soil contracts, weakens, and cracks. During wet seasons, the cracks provide paths for infiltration and subsurface flows. The latter dissolve, chemically removing soil fractions and horizontally transporting minerals in solution with flowing water and then vertically to the surface through leaching.

Tunneling and gullying also occur in formations in low- and flat-lands that contain marls and silts. Erosion begins along gully scarps where water may stagnate and create holes from shrink and swell processes. As a hole grows, it may eventually connect to a main gully, widening and elongating it. Piping, tension-crack development, dispersion, bank collapse, and rill erosion are important mechanisms initiating and developing gullies.

## 3. Materials and Methods

### 3.1. Data Used

**Figure 2:**
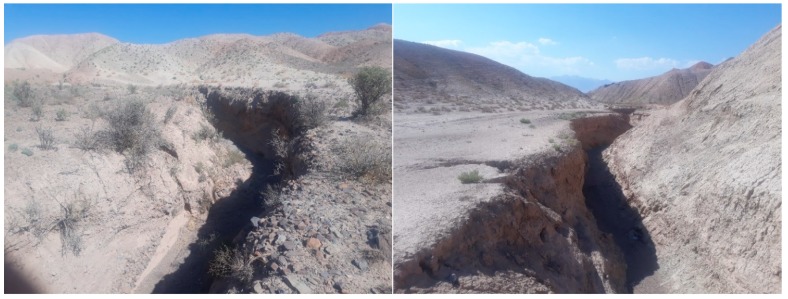
Sample of gullies in the study area.

A gully-erosion inventory map (GEIM) was developed from an extensive field survey. Three hundred and six gully erosion events were identified in the region and geolocated with a global positioning system (GPS) device (Figure 2). They were randomly divided into a training set (70%, 213 gully locations) and a validation set (30%, 92 gully locations). Gullies occupy 141.3 km^2^, comprising 5% of the study area. The gullies, mapped as polygons, were converted to points (locations of the head-cut portion of each gully). The point locations were used in modeling and validation. An equivalent number and percentage of non-gully point locations were randomly chosen and were used in calibration and validation procedures.

**Figure 3:**
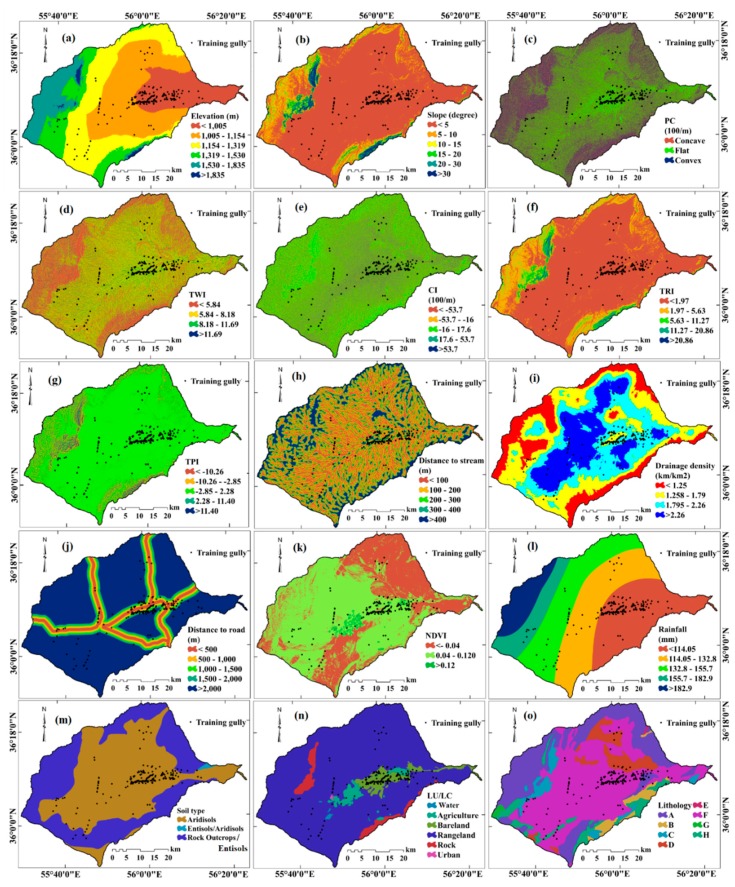
Gully erosion conditioning factors. (**a**) elevation, (**b**) slope, (**c**) plan curvature (PC), (**d**) topography wetness index (TWI), (**e**) convergence index (CI), (**f**) Terrain Ruggedness Index (TRI), (**g**) topography position index (TPI), (**h**) distance to stream, (**i**) drainage density, (**j**) distance to road, (**k**) Normalized Difference Vegetation Index (NDVI), (**l**) rainfall, (**m**) soil type, (**n**) land use/land cover (LU/LC), (**o**) lithology.

The fifteen most important gully erosion conditioning factors (GECFs) were chosen based on a literature review [3,11,13,14,15], assessments of the physical characteristics of the study area, the scale, and availability of data, and a consideration of the intended purpose of research. These GECFs are elevation, slope, plan curvature (PC), topography wetness index (TWI), convergence index (CI), terrain ruggedness index (TRI), topography position index (TPI), distance to stream, drainage density, distance to road, normalized difference vegetation index (NDVI), rainfall, soil type, land use/land cover (LU/LC), and lithology (Table 1). An ALOS DEM with a spatial resolution of 12.5 m, topography and geology maps with 1:50,000 (www.ngo-org.ir) and 1:100,000 (Toroud sheet) scales, LANDSAT-8 images archived by USGS (https://earthexplorer.usgs.gov/), a soil map with 1:100,000 scale, and rainfall statistics for a 30-year period (1986 to 2016) were used to prepare GECFs (Figure 3a–o).

Elevation affects vegetation and precipitation patterns. They therefore control the spatial distribution of gully erosion and the processes at work [36]. Slope affects surface runoff, soil erosion, and drainage density patterns. The steepness of the slope is also important as it enhances or attenuates the energy of erosive processes and gully erosion [37]. Plan curvature causes convergence or divergence of water flows on slopes and influences downslope flow [36]. These three parameters were extracted from the ALOS DEM.

Erosive-runoff capacity reflects transport capacity and flow velocity, and is determined by TWI. TWI is crucial for identifying areas prone to gully erosion [36]. The CI measures how flow in a cell diverges (negative CI values) or converges (positive CI values) [38]. The TRI indicates convexity and concavity of slopes which influences gully erosion [39]. The TPI compares the height of each pixel in the DEM to the average height of the pixels around it. This factor enables classification of landscapes into morphological classes. Positive and negative values indicate that a pixel is higher or lower in elevation than the pixels that surround it [40]. TWI, SPI, TRI, and TPI were calculated with Equations (1)–(4) [41,42]:

(1) $$TWI=In\;(A_{S}/tan\beta ,$$

(2) SPI=As×tanσ,

(3) $$TRI=\sqrt{\left|X\right|\left(max^{2}-min^{2}\right)},$$

(4) $$TPI=\frac{E_{\mathrm{pixel}}}{E_{\mathrm{surrounding}}},$$

where $A_{S}$ is the catchment area of the basin (m^2^/m), *β* is slope steepness (degrees), *x* is the elevation of each neighbor cell to a specific cell (0,0) (m), and max and min are the largest and smallest elevations among the nine neighboring pixels. E_pixel_ is the elevation of the cell, and E_surrounding_ is the mean elevation of the neighbor pixels.

Gully erosion depends on the lithology of the material at or near the surface [43,44]. The lithology layer was prepared by digitizing a geological map (Geological Survey Department of Iran, Toroud sheet at 1:100,000 scale) [34]. Description of lithology units in the study area are shown in Table S1.

Land use/land cover (LU/LC) impacts slope stability and gully formation. Bare lands are very prone to erosion, but land with vegetative cover has significantly less erosion [45]. An LU/LC map of the study area was produced from Landsat 8 imagery sensed on 9 August 2018. A supervised classification using the maximum likelihood algorithm was used to create the LU/LC map. The map was ground-truthed using 495 ground control points (GCP). The Kappa coefficient of the map generated is 96.23%. Gullies are often linked to the drainage/stream network, and they facilitate the transport of material eroded from upland areas [36]. Higher drainage densities generate greater amounts of surface runoff [46]. To measure the parameter distance to stream and drainage density, the stream network was extracted from a PALSAR DEM in Arc Hydro. To provide more accurate flow direction and flow accumulation measures, holes in the DEM were filled, after which flow direction and accumulation were extracted. A threshold of 500 cells was used to extract the stream network. After stream extraction, the Euclidean Distance and Line Density tools in ArcGIS10.5 were used to calculate distances and densities of streams.

Roads as impenetrable surfaces disrupt natural drainage with improperly constructed culverts, by concentrating surface runoff, and by altering the hydrological functions of hillslopes. These impacts significantly increase overland flow and enable more rapid run-off, which easily erodes bare soil and causes gullying [47]. The distance to road measurements were computed from a road network layer that was extracted from a 1:50,000-scale topographic map and Google Earth images. Vegetation protects soil from many types of erosion. Vegetation can decrease the vulnerability of an area by aiding infiltration and holding soil in places with plants’ roots [2]. The NDVI was computed from LANDSAT-8 data and Equation (5):

(5) NDVI=IR−R/IR+R,

where *IR* is the infrared portion of the electromagnetic spectrum and *R* is the red portion of the electromagnetic spectrum. The layers were unified using the UTM Zone39N geographic coordinate system at a pixel size of 12.5 m (the DEM’s spatial resolution). The classes of the GECFs are presented in Table 2.

### 3.2. Background of the Methods Used

#### 3.2.1. Frequency Ratio (FR) and Statistical Index (SI)

Two bivariate statistical models, FR and SI (descriptions and explanations found in [53,54,55,56]), have high potential for modeling environmental processes [53]. In these models, GECF and GEIM are considered dependent and independent variables, respectively. Each GECF thematic layer was analyzed relative to gullying to generate a weight of classes for that factor. The probability of gullying for each pixel was calculated using the algebraic sum of the weights of classes of all layers.

#### 3.2.2. Random Forest (RF)

The RF model can be used to assess environmental issues and hazards [57]. This model combines several tree algorithms to generate repeated predictions of each phenomenon [58]. It can also learn complicated patterns and factor in the nonlinear relationships between explanatory and dependent variables. It can also incorporate and combine different data types because it does not assume anything about the distributions of the data. This model can incorporate thousands of input variables without deleting any. Details of RF can be found in [59]. In this study, RF analyses were conducted in R 3.3.1 using the ‘Randomforest’ package [60].

#### 3.2.3. Maximum Entropy (ME)

ME is a prediction model guided by entropy maximization [61]. This model maximizes the probabilities without parametric assumptions about the input variables [62]. A detailed explanation of ME can be found in [63].

#### 3.2.4. Generalized Linear Model (GLM)

GLM is the extension of the classic linear-regression model [64]. A detailed explanation of this model can be found in [64]. The species distribution modeling (SDM) package [65] was used to run GLM in R 3.3.3.

#### 3.2.5. Functional Data Analysis (FDA)

The FDA model, suitable for observation data consisting of a series of real functions, was proposed by Ramsay and Dalzell [66]. A detailed explanation of the FDA model can be found in [67]. The FDA model was used to construct the GESM with the SDM package in R [65].

#### 3.2.6. Technique for Order Preference by Similarity to Ideal Solution (TOPSIS)

TOPSIS was introduced by Hwang and Yoon [68]. The underlying logic of TOPSIS is to define the positive and negative ideal solutions. Details of this model are found in [69]. To prepare GESM with TOPSIS, the qualitative parameters were converted into quantitative parameters using the FR method. The parameters should have ascending or descending trends. Parameters that did not follow either of these trends were also weighted using the FR method. Once weighted, GECF values for 500 randomly selected points in the study area were extracted and input into SPSS. A decision matrix with 15 columns and 500 rows was prepared. The TOPSIS model was then applied in SPSS and the final weight of each point was determined. The GIS point layer was populated using interpolation (kriging), thus creating a GESM.

#### 3.2.7. Ensemble Approaches (GLM–FDA, FR–RF and SI–RF)

The consensus is that each model has its apparent advantages and disadvantages [3]. In this study, an ensemble of five models—FR, SI, GLM, FDA and RF—were used to produce GESMs. These integrated methods eliminate several disadvantages of bivariate methods: the failure to calculate the importance of parameters and non-calculation of the spatial relationships between the feature of interest (e.g., gullying) and the parameters that affect their formation.

### 3.3. Methodology

**Figure 4:**
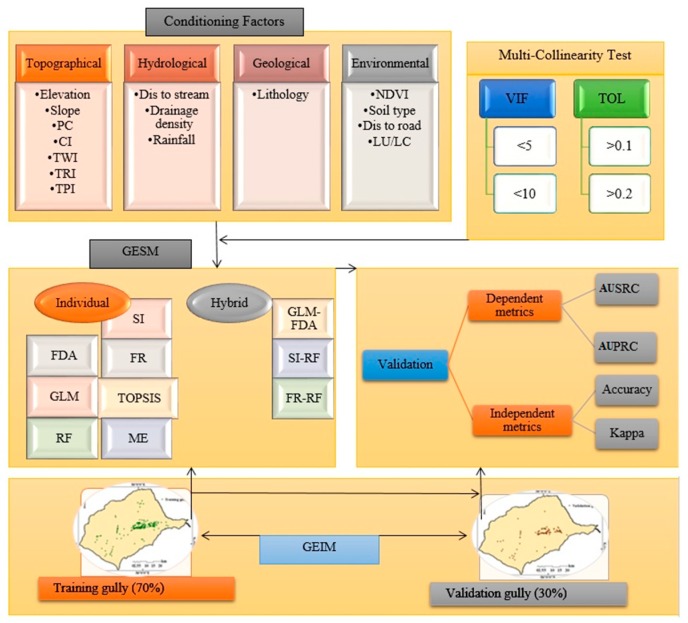
Flowchart of research in the study area.

This research consists of several main steps (Figure 4). Data collection occurred either in the library, in the field, or in the laboratory.

Step 1: Database preparation.
Step 2: Multicollinearity analysis. If collinearity occurs among the parameters, the prediction accuracy of a model will decrease [3]. Indices of tolerance (TOL) and variance inflation factor (VIF) were used to evaluate collinearity [70]. If VIF ≤ 5 or 10 and TOL≤ 0.1 or 0.2, then no collinearity exists between factors [71].
Step 3: Configuring and training the GE models.
Step 4: Performance assessment using cutoff dependence (Area under prediction rate curve [AUPRC] and area under success rate curve [AUSRC]) and cutoff independence (accuracy and kappa).
Step 5: GESM generation.

## 4. Results

### 4.1. Multicollinearity Test (MT)

The MT (Table 2) showed that no collinearity existed amongst conditioning factors. The minimum and maximum of TOL and VIF were (0.214–0.940) and (1.108–4.66), respectively. All thematic layers were used in the modeling processes.

### 4.2. Spatial Relationship between Conditioning Factors and Gully Locations

The spatial relationships between gully locations and the GECFs calculated with the FR and SI models are shown in Table 3. Elevations below 1005 m, slopes < 5°, flat plan curvature, TWI > 11.69, and CI > 53.7 are strongly correlated with gullies. Regarding TRI < 1.09, TPI from −2.85 to 2.28, <100 m from a stream, drainage densities ranging from 1.79 to 2.26 km/km^2^, and places between 500 to 1000 m to the nearest road were the most susceptible to GE. NDVI between −0.04 and 0.12, <114.05 mm rain, aridisols, bareland (LU/LC) and Quaternary lithotypes (clayey material, well-sorted sand dunes, salt concretions, mixed terrace deposits and swamp or marsh deposits) were strongly correlated with gullies.

### 4.3. Relative Importance of Conditioning Factors Using the RF Model

**Figure 5:**
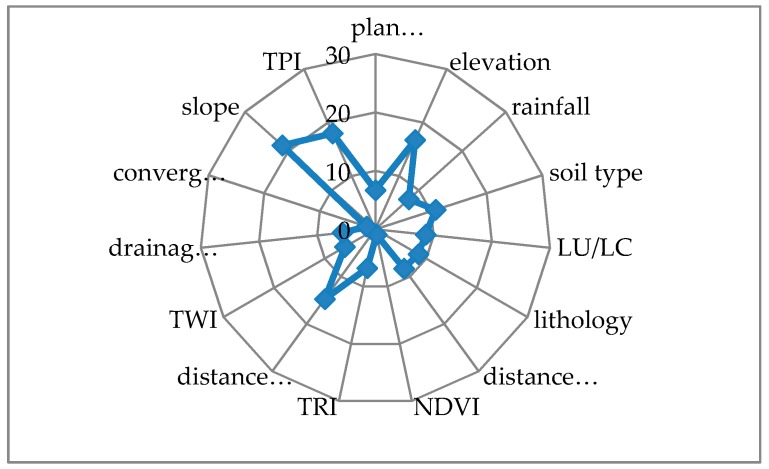
Relative importance of gully erosion conditioning factors using the random forest model.

The relative importance of conditioning factors was determined using the RF model (Figure 5). Slope (21.46), TPI (17.96), and elevation (16.89) were keys to GE in the study area. By contrast, NDVI, convergence index, and drainage density are least important determinants of gully formation. The distance to stream, soil type, LU/LC, lithology, distance to road, rainfall, TRI, plan curvature, and TWI rank from 4th to 12th, respectively.

### 4.4. Gully Erosion Susceptibility Mapping (GESM)

**Figure 6:**
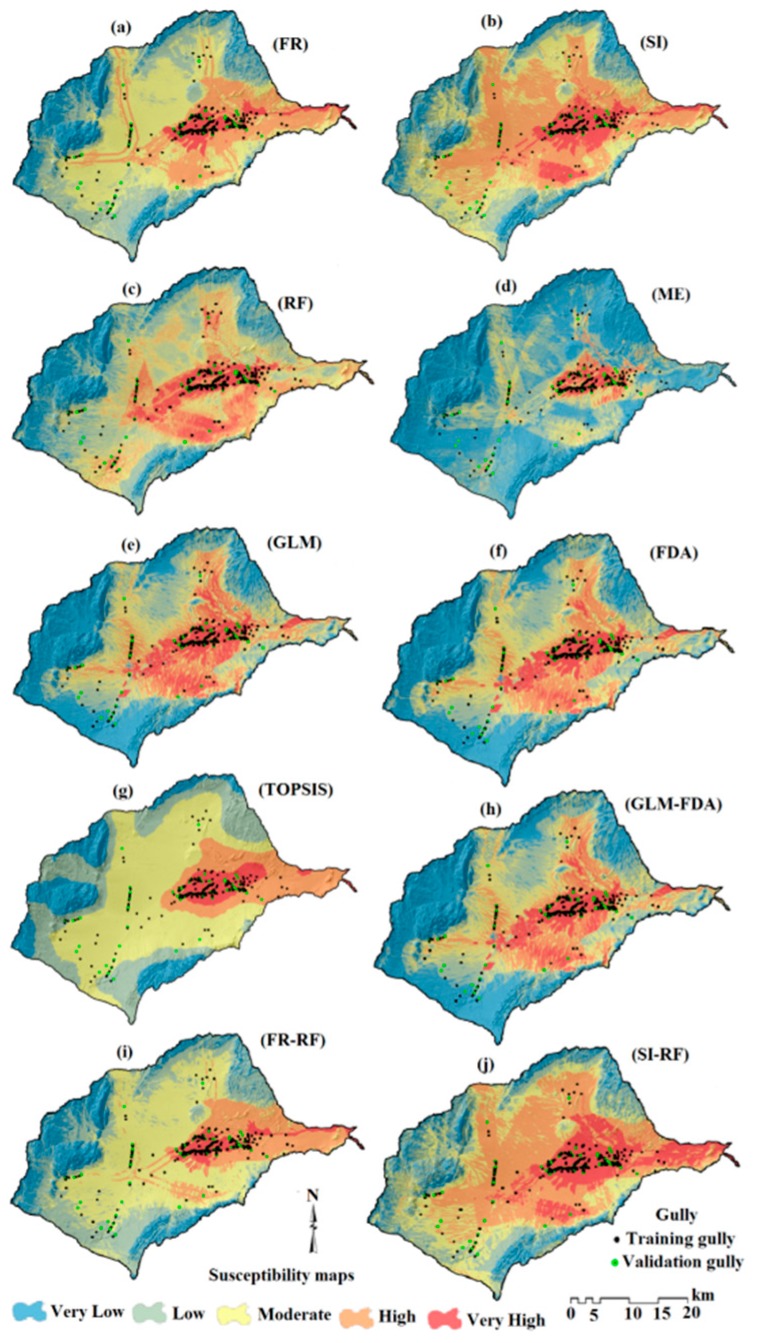
Gully erosion susceptibility maps. (**a**) frequency ration (FR), (**b**) statistical index (SI), (**c**) random forest (RF), (**d**) maximum entropy (ME), (**e**) generalized linear model (GLM), (**f**) functional data analysis (FDA), (**g**) TOPSIS, (**h**) GLM–FDA, (**i**) FR–RF, (**j**) SI–RF.

**Figure 7:**
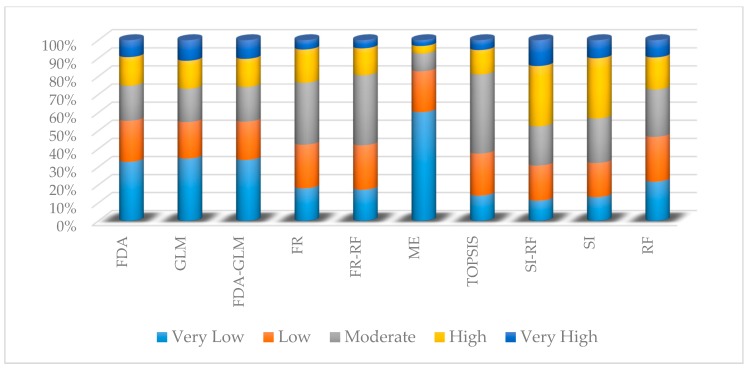
Percentage of each susceptibility classes in individual and hybrid models.

The minimum and maximum values (Table 4) of the GESMs (Figure 6a–j) produced with the 10 models are diverse. The proportions of the study area classified into the five susceptibility classes by each model (Figure 7) display significantly different results, with SI–RF and SI generating the most widespread classification of land into high or very high susceptibility and ME classifying the greatest percentage of the area as low or very low. 

### 4.5. Validation of Results

**Figure 8:**
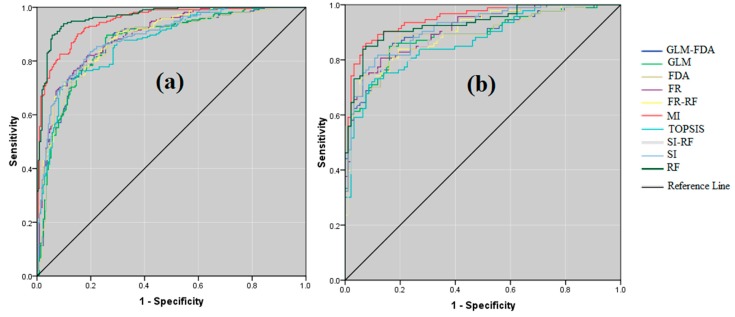
Area under curve in individual and ensemble models. (**a**) training data (success rate curve), (**b**) validation data (prediction rate curve).

**Figure 9:**
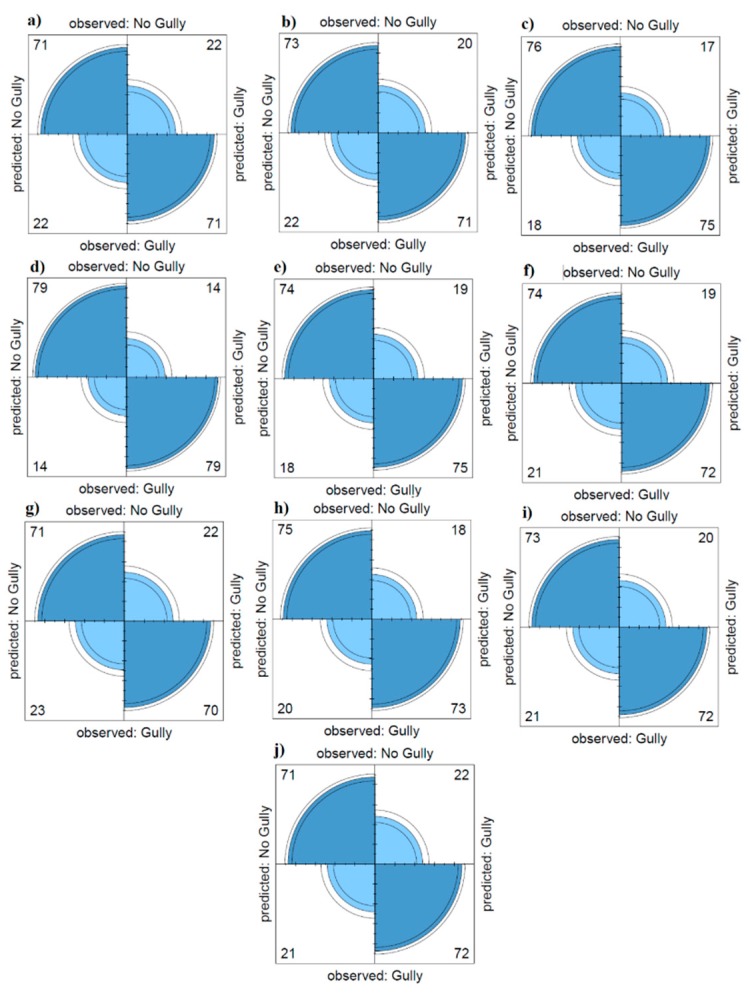
Values of true negative (TN), false positive (FP), true positive (TP) and false negative (FN) for each individual or ensemble model. (**a**) FR, (**b**) SI, (**c**) RF, (**d**) ME, (**e**) GLM, (**f**) FDA, (**g**) TOPSIS, (**h**) GLM–FDA, (**i**) FR–RF, (**j**) SI–RF.

Validation employed AUSRC (Figure 8) and revealed the RF model (AUSRC = 0.965) (Table 5) performed best, whereas considering AUPRC, accuracy, and kappa criteria, the ME model (AUPRC = 0.948, accuracy = 0.849 and kappa = 0.699) performed best, followed by RF (Table 5 and Figure 9). Results indicate that combining the RF model with FR and SI models increased the performances of the latter compared to them as stand-alone models.

## 5. Discussion

In this study, 10 GIS-based statistical, machine-learning, and multicriteria models were integrated with RS data to generate GESMs. The statistical models FR and SI were included because of their simplicity and high efficiency, and to ease of interpretation of the results [72,73,74]. The TOPSIS method was used because it requires only simple calculations and minimal computation time, has the capacity to rank the alternatives, uses both quantitative and qualitative criteria, and can determine the relative importance of alternatives and compliance with the conditions. The TOPSIS method, being local and experimental, is known as one of the best methods for decision making [75]. It is well suited for several scenarios and criteria [67,75]. The RF, MI, FDA, and GLM models were included because of their capacities to predict environmental phenomena [63,66]. In cases in which key data are missing, the FDA model is more efficient than traditional methods. The GLM method solves nonlinear and multiclass problems well [63]. Any of the models in this set of selected models could produce a reliable GESM.

Assessments of the spatial relationships between the GECFs and the gullies show that gullies formed mainly in areas below 1005 m elevation and on slopes angles less than 5°. Topography affects vegetation types, drainage area, geomorphological processes, weathering, soil moisture, drainage density, soil types, and precipitation. All these directly or indirectly influence GE potential [17,76,77]. Areas with gentle slopes have a high potential for accumulation of overland flows that can initiate gullying [78]. Surface and subsurface water are also key factors for gullying [79]. In both circumstances (above and below ground), the slope is the main factor initiating gully formation [80].

Flat topography is highly correlated with susceptibility to GE. This agrees with the findings of [51]. High TWI is strongly correlated with gullies in the study area. Arabameri et al. [72] used an integrated model to predict GE in the Mahabia watershed and also stated that areas with high TWI also had high positive susceptibility. Drainage density is highly correlated with susceptibility to GE, confirming the findings of [19,81,82]. Proximity to rivers and roads was positively correlated to gullies, as was reported by [11,83,84]. Natural drainage patterns are often disrupted by poorly located or poorly constructed culverts placed during road construction. Subsequently, soil is easily eroded by concentrated runoff created by impervious surfaces [85].

NDVI analysis reveals that areas with more vegetation growth had fewer gullies; areas with less vegetation had higher frequency of gully formation. This finding corroborates the results of [5,11]. Vegetation greatly reduces runoff and limits erosion by increasing infiltration and by protecting soil with root growth [86,87]. Aridisols are highly susceptible to GE in this region, and this agrees with [88].

Land use underpins geomorphological and hydrological processes by affecting runoff generation, sediment dynamics, and overland flows [89]. LU/LC analysis showed that agricultural and bare landscapes, where soil is often disturbed, where surface water is often concentrated [90,91,92,93], and where the surface is often unprotected by vegetation [88], had the highest susceptibility to GE in our study region. Because GE depends on the lithology of materials at or just below the surface [5] the spatial patterns of sediment origins were evaluated. Quaternary lithotypes in the study area have the highest susceptibility to GE, which is coincident with the findings of [88]. Gullying is a natural phenomenon that depends on the thresholds of several conditioning factors (e.g., rainfall, topography, flow hydraulics, pedology and land use). It is more likely to occur at locations where thresholds have been exceeded [94].

Examination of the relative importance of GECFs shows that slope, TPI, and elevation were the most important in the study area and corroborates [11,12,13,23]. Zabihi et al. [13] tested three models (FR, WoE and IoE) to model GE in Iran and found that of 12 GECFs, elevation and LU/LC were the most important in their study area. Meliho et al. [12] used IV and FR for GESM in the Ourika watershed in Morocco, and they found that LU/LC and slope had the most influence on gully formation.

The GESMs were classified into five different gully-erosion susceptibility classes (very low to very high) using four classification methods: geometrical interval, quantile, equal interval, and natural breaks. Comparing the results of each classification method with the high and very high gully-erosion susceptibility classes, it is clear that the natural break method provides the most accurate classification scheme. This agrees with the findings of [3].

The validation results using cutoff-dependent (AUPRC and AUSRC) and cutoff-independent (accuracy and kappa) criteria shows that ML models outperform statistical and MCDM-based models [19,70,88,95]. ML models are advantageous because they do not require a strict set of assumptions as is the case with many statistical methods [22]. ML models also use algorithms to discern the relationships between GECFs and gullies and therefore do not rely on a structural model [19]. These models also benefit from iterative learning algorithms which help them learn and improve [19].

AUSRC shows that RF had the best performance among the 10 models. This confirms the findings of [88,96]. Arabameri et al. [88] used three data-mining models for gully-erosion assessment in the Shahroud watershed in northeastern Iran, and found that RF performed best. When there is considerable noise in data, this method is less sensitive to ANNs and can better assess factors compared with others [97,98]. The most important advantages of RF models are their capacities to learn nonlinear relationships, that they have high predictive accuracy, they are able to determine relative factor importance, they can deal with distorted data, and they have high categorization ability.

Based on the AUPRC, accuracy, and kappa results, ME was shown to have the best overall performance. This reflects similar findings in [98,99,100,101]. ME exceeds other ML models because it uses search-based optimization to determine the relative importance of factors [101]. Results of the ensemble RF–FR and RF–SI statistical models reveal that RF is one of the best classification algorithms to use to considerably improve the performance of single classifiers [102,103]. Moreover, RF can decrease the dependence of statistical models on the relationships among the conditioning factors [30].

## 6. Conclusions

GE is a bane of rural sectors in arid and semi-arid regions of the world. To combat the formation of gullies, it is often necessary to diagnose the scope of the problem and the causes endemic to the local environment. GESM is a key tool for sustainable management and use of soil and water resources. The foundation of GESM is the spatial predictive model. There is no consensus among scholars about the best modeling approach (statistical, ML, or MCDM) to generate an accurate spatial assessment of GE. There are constant technological advancements that make data available for incorporation and analysis in ways that are more efficient and economical. These conditions are very important in regions that are more remote or that have fewer financial resources. This study attempted to tackle this question by comparing ten individual and ensemble models—FR, SI, RF, ME, FDA, GLM, TOPSIS, SI–RF, FR–RF and GLM–FDA—from among the extant statistical, ML, and MCDM approaches to model gully-erosion susceptibility in the Dasjard River watershed, Iran. RS data and GIS techniques were used to compile and analyze 15 environmental, geological, geomorphological, and anthropogenic GECFs that were selected according to the MT. VIF and TOL indicate that there is no multicollinearity among them. The results show that the RF ML model performed the best according to the AUROC. In this model, slope, TPI, and elevation were the key factors generating gullying in the study area. The RF model combines several tree algorithms to iteratively predict a phenomenon. RF can learn complicated patterns and can consider nonlinear relationships between dependent and independent variables. Furthermore, it can integrate data of different types, requires no assumptions about the normality of the data used, and can incorporate thousands of variables without discarding any of them. Based on the AUSRC, AUPRC, accuracy, and kappa values, ME outperformed even RF. Validation tests indicate that RF and ME, which are both ML models, outperformed all statistical and MCDM models tested. Ensemble models, particularly the combinations of RF with FR and SI, improved the prediction accuracy and success achieved by the individual models on their own. The scientific achievement of this study is that, by combining ML models with a suitable set of GECFs, data describing extant gullies, RS data, and GIS, one can produce reliably accurate GESMs. These GESMs were achieved with a method that is easy to use and can provide valuable information for planners or managers to prevent or respond to gully-erosion problems. This methodology can be used to assess gully-erosion susceptibility in similar regions of the world, especially in arid and semi-arid environments.

## Figures and Tables

**Table 1:** Classes and classification method for the various thematic data layers.

No.	Factor	Classes	Classification Method	References
1	Elevation (m)	1. <1005, 2. 1005–1154, 33. 1154–1319, 4. 1319–1530, 5. 1530–1835, 6. >1835	Natural break	[48]
2	Slope (°)	1. <5, 2. 5–10, 3. 10–15, 4. 15–20, 5. 20–30, 6. >30	Manual	[49]
3	Plan curvature (m^−1^)	1. Concave, 2. Flat, 3. Convex	Manual	[48]
4	TWI	1. <5.84, 2. 5.84–8.18, 3. 8.18–11.69, 4. >11.69	Natural break	[48]
5	CI	1. <–53.7, 2. −53.7–−16, 3. −16–17.6, 4. 17.6–53.7, 5. >53.7	Natural break	[48]
6	TRI (m)	1. <1.97, 2. 1.97–5.63, 3. 5.63–11.27, 4. 11.27–20.86, 5. >20.86	Natural break	[50]
7	TPI	1. <−10.26, 2. −10.26–−2.85, 3. −2.85–2.28, 4. 2.28–11.4, 5. > 11.4	Natural break	[50]
8	Distance to river (m)	1. <100, 2. 100–200, 3. 200–300, 4. 300–400, 5. >400	Manual	[48]
9	Drainage density (km/km^2^)	1. < 1.25, 2. 1.25–1.79, 3. 1.79–2.26, 4. >2.26	Natural break	[51]
10	Distance to road (m)	1. <500, 2. 500–1000, 3. 1000–1500, 4. 1500–2000, 5. >2000	Manual	[52]
11	NDVI	1. <−0.04, 2. −0.04–0.12, 3. >0.12	Natural break	[48]
12	Rainfall	1. <114.05, 2.114.05–132.8, 3. 132.8–155.7, 4. 155.7–182.9, 5. <182.9	Natural break	[48]
13	Soil	1. Rock Outcrops/Entisols, 2. Aridisols, 3. Entisols/Aridisols	Soil type	
14	LULC	1. Abkhan, 2. Agriculture, 3. Bareland, 4. Rangeland, 5. Rock, 6. Urban	Land use type	
15	Lithology	1. A, 2. B, 3. C, 4. D, 5. E, 6. F, 7. G, 8. H	Lithology type	

**Table 2:** Multi-collinearity analysis among gully erosion conditioning factors.

* Factors	Unstandardized Coefficients		Standardized Coefficients	t	Sig.	Collinearity Statistics	
	B	Std. Error	Beta			Tolerance	VIF
(Constant)	−0.272	0.164		−1.666	0.096		
lithology	−0.006	0.038	−0.007	−0.159	0.874	0.554	1.805
LU/LC	0.016	0.005	0.164	3.033	0.003	0.415	2.412
Soil type	0.083	0.037	0.104	2.205	0.028	0.546	1.831
Drainage density	0.073	0.029	0.107	2.517	0.012	0.672	1.489
Rainfall	0.002	0.029	0.005	0.076	0.939	0.245	4.078
Slope	0.143	0.110	0.092	1.296	0.196	0.241	4.153
TRI	−0.079	0.108	−0.055	−0.729	0.466	0.214	4.668
TPI	0.048	0.079	0.027	0.601	0.548	0.586	1.706
TWI	−0.082	0.059	−0.055	−1.404	0.161	0.784	1.276
PC	0.054	0.106	0.019	0.506	0.613	0.903	1.108
NDVI	0.102	0.040	0.107	2.526	0.012	0.685	1.459
Dis to stream	0.126	0.041	0.116	3.084	0.002	0.863	1.158
Dis to road	0.080	0.013	0.263	6.148	0.000	0.666	1.501
elevation	0.055	0.020	0.202	2.815	0.005	0.237	4.224
CI	−0.070	0.085	−0.029	−0.818	0.414	0.940	1.063

* LU/LC: land use/land cover, TRI: Terrain Ruggedness Index, TPI: topography position index, TWI: topography wetness index, PC: plan curvature, NDVI: Normalized Difference Vegetation Index, CI: convergence index.

**Table 3:** Spatial relationship between conditioning factors and gully locations using frequency ratio and statistical index.

* Factor	Class	Pixels in Domain		Gullies		FR	SI
		No	%	No	%		
Elevation (m)	<1005	409583	16.47	143	67.45	4.09	1.41
	1005–1154	778,768	31.32	45	21.23	0.68	−0.39
	1154–1319	675,092	27.15	17	8.02	0.30	−1.22
	1319–1530	314,348	12.64	7	3.30	0.26	−1.34
	1530–1835	290,378	11.68	0	0.00	0.00	None
	>1835	18,056	0.73	0	0.00	0.00	None
Slope (°)	<5	2,018,483	81.19	205	96.70	1.19	0.17
	5–10	235,497	9.47	7	3.30	0.35	−1.05
	10–15	98,979	3.98	0	0.00	0.00	None
	15–20	46,006	1.85	0	0.00	0.00	None
	20–30	44,839	1.80	0	0.00	0.00	None
	>30	42,421	1.71	0	0.00	0.00	None
PC (100/m)	Concave	792,994	31.90	55	25.94	0.81	−0.21
	Flat	907,578	36.50	94	44.34	1.21	0.19
	Convex	785,652	31.60	63	29.72	0.94	−0.06
TWI	<5.84	805,518	32.40	33	15.57	0.48	−0.73
	5.84–8.18	1,120,812	45.08	123	58.02	1.29	0.25
	8.18–11.69	408,848	16.44	38	17.92	1.09	0.09
	>11.69	151,046	6.08	18	8.49	1.40	0.33
CI (100/m)	<−53.7	170,770	6.98	16	7.55	1.08	0.08
	−53.7–−16	614,268	25.10	44	20.75	0.83	−0.19
	−16–17.6	994,363	40.63	83	39.15	0.96	−0.04
	17.6–53.7	535,208	21.87	54	25.47	1.16	0.15
	>53.7	133,049	5.44	15	7.08	1.30	0.26
TRI	<1.97	1,995,829	80.28	207	97.64	1.22	0.20
	1.97–5.63	314,118	12.63	5	2.36	0.19	−1.68
	5.63–11.27	120,287	4.84	0	0.00	0.00	None
	11.27–20.86	45,815	1.84	0	0.00	0.00	None
	>20.86	10,176	0.41	0	0.00	0.00	None
TPI	<−10.26	27,479	1.11	0	0.00	0.00	None
	−10.26–−2.85	202,970	8.16	5	2.36	0.29	−1.24
	−2.85–2.28	2,103,205	84.59	207	97.64	1.15	0.14
	2.28–11.4	130,891	5.26	0	0.00	0.00	None
	>11.4	21,679	0.87	0	0.00	0.00	None
Dis to stream (m)	<100	881,433	35.45	117	55.19	1.56	0.44
	100–200	625,868	25.17	54	25.47	1.01	0.01
	200–300	443,260	17.83	25	11.79	0.66	−0.41
	300–400 > 400	224,458 311,205	9.03 12.52	10 6	4.72 2.83	0.52 0.23	−0.65 −1.49
Drainage density (km/km^2^)	<1.25	461689	18.57	2	0.94	0.05	−2.98
	1.25–1.79	746549	30.03	55	25.94	0.86	−0.15
	1.79–2.26	712235	28.65	124	58.49	2.04	0.71
	>2.26	565752	22.76	31	14.62	0.64	−0.44
Dis to road (m)	<500	177023	7.12	32	15.09	2.12	0.75
	500–1000	168791	6.79	68	32.08	4.72	1.55
	1000–1500	159125	6.40	31	14.62	2.28	0.83
	1500–2000	151080	6.08	26	12.26	2.02	0.70
	> 2000	1830206	73.61	55	25.94	0.35	−1.04
NDVI	<−0.04	918021	36.92	17	8.02	0.22	−1.53
	−0.04–0.12	1541694	62.01	192	90.57	1.46	0.38
	>0.12	26510	1.07	3	1.42	1.33	0.28
Rainfall (mm)	<114.05	688309	27.68	161	75.94	2.74	1.01
	114.05–132.8	694011	27.91	21	9.91	0.35	−1.04
	132.8–155.7	619724	24.93	23	10.85	0.44	−0.83
	155.7–182.9	259107	10.42	7	3.30	0.32	−1.15
	<182.9	225074	9.05	0	0.00	0.00	None
Soil type	Rock Outcrops/Entisols	1035170	41.64	12	5.66	0.14	−2.00
	Aridisols	1443591	58.06	199	93.87	1.62	0.48
	Entisols/Aridisols	7464	0.30	1	0.47	1.57	0.45
LU/LC	Abkhan	5419	0.22	0	0.00	0.00	None
	Agriculture	73837	2.97	11	5.19	1.75	0.56
	Bareland	124293	5.00	124	58.49	11.70	2.46
	Rangeland	2182822	87.80	76	35.85	0.41	−0.90
	Rock	98132	3.95	1	0.47	0.12	−2.12
	Urban	1722	0.07	0	0.00	0.00	None
Lithology	A	697041	28.04	20	9.43	0.34	−1.09
	B	79375	3.19	1	0.47	0.15	−1.91
	C	114582	4.61	3	1.42	0.31	−1.18
	D	190539	7.66	10	4.72	0.62	−0.49
	E	2747	0.11	0	0.00	0.00	None
	F	153705	6.18	4	1.89	0.31	−1.19
	G	1236071	49.72	174	82.08	1.65	0.50
	H	12165	0.49	0	0.00	0.00	None

* LU/LC: land use/land cover, TRI: Terrain Ruggedness Index, TPI: topography position index, TWI: topography wetness index, PC: plan curvature, NDVI: Normalized Difference Vegetation Index, CI: convergence index.

**Table 4:** Values of resulted gully erosion maps using ten models.

* Models	Classification with a Natural Break Model				
	Very Low	Low	Moderate	High	Very High
FR	3.66–9.63	9.63–13.79	13.79–17.96	17.96–26.57	26.57–39.06
SI	−19.3–−10.5	−10.5–−6	−6–−2.27	−2.27–2.25	2.25–10.25
RF	0.01–0.21	0.21–0.37	0.37–0.53	0.53–0.72	0.72–1
ME	0.00–0.06	0.06–0.17	0.17–0.34	0.34–0.57	0.57–0.97
GLM	0.00–0.12	0.12–0.3	0.3–0.49	0.49–0.69	0.69–0.98
FDA	0.00–0.13	0.13–0.31	0.31–0.51	0.51–0.73	0.73–0.99
TOPSIS	0.15–0.29	0.29–0.38	0.38–0.48	0.48–0.61	0.61–0.78
GLM-FDA	0.00–0.13	0.13–0.31	0.31–0.5	0.5–0.71	0.71–0.99
FR-RF	21.72–84.7	84.7–131.2	131.2–183.2	183.2–268.1	268.1–370.8
SI-RF	−190–−99.9	−99.9–−59.3	−59.3–−23.2	−23.2–18.4	18.4–97.4

* FR: frequency ratio, SI: statistical index, RF: random forest, ME: maximum entropy, GLM: generalized linear model, FDA: functional data analysis, TOPSIS: Technique for order preference by similarity to ideal solution.

**Table 5:** Validation of gully erosion susceptibility maps using cutoff-dependent and independent criteria.

	* Criteria	TN	FP	FN	TP	TPR	TNR	FPR	Cutoff-Dependent Criteria		Cutoff Independent Criteria	
* Models									AUSRC	AUPRC	Aaccuracy	Kappa
FR		71	22	22	71	0.76	0.76	0.23	0.890	0.900	0.763	0.527
SI		73	20	22	71	0.76	0.78	0.21	0.884	0.897	0.774	0.548
RF		76	17	18	75	0.80	0.81	0.18	0.965	0.932	0.812	0.624
ME		79	14	14	79	0.84	0.84	0.15	0.947	0.948	0.849	0.699
GLM		74	19	18	75	0.80	0.79	0.20	0.869	0.887	0.801	0.602
FDA		74	19	21	72	0.77	0.79	0.20	0.868	0.894	0.785	0.570
TOPSIS		71	22	23	70	0.75	0.76	0.23	0.871	0.867	0.758	0.516
GLM-FDA		75	18	20	73	0.78	0.80	0.19	0.870	0.891	0.796	0.591
FR-RF		73	20	21	72	0.77	0.78	0.21	0.893	0.908	0.780	0.559
SI-RF		71	22	21	72	0.77	0.76	0.237	0.889	0.914	0.769	0.538

* FR: frequency ratio, SI: statistical index, RF: random forest, ME: maximum entropy, GLM: generalised linear model, FDA: functional data analysis, TOPSIS: Technique for order preference by similarity to ideal solution; * TN: true negative, FP: false positive, TP: true positive, FN: false negative, TNR: true negative rate, FPR: false positive rate, AUSRC: area under success rate curve, AUPRC: area under prediction rate curve.

## Supplementary Materials

The following are available online at https://www.mdpi.com/1424-8220/20/2/335/s1, Table S1: Lithology of study area.
