# Supplementary material for: Evaluation of Recent Advanced Soft Computing Techniques for Gully Erosion Susceptibility Mapping: A Comparative Study
Source: Sensors (Basel). 2020 Jan 7;20(2):335. doi: 10.3390/s20020335 (PMC7014250; doi:10.3390/s20020335)
Supplement: Supplementary file 1 [file sensors-20-00335-s001.pdf]

Lithology of study area

Group	Code	Descriptiom
A	E2l	Nummulitic limestone
	Ea.bvt	Andesitic to basaltic volcanic tuff
	E3sm	Sandstone and marl
	Eat	Andesitic tuff
	E1m	Marl, gypsiferous marl and limestone
	E2c	Conglomerate and sandstone
	E1c	Pale-red, polygenic conglomerate and sandstone
	E1f	Silty shale, sandstone, marl, sandy limestone, limestone and conglomerate
	E1s	Sandstone, conglomerate, marl and sandy limestone
	Eavt	Andesitic volcanic tuff
B	Jbg	Pale - green silty shale and sandstone
	Jugr	Upper Jurassic granite including Shir Kuh Granite and Shah Kuh Granite
	Jd	Well - bedded to thin - bedded, greenish - grey argillaceous limestone with intercalations of calcareous shale
	Je	Massive, light-grey reef limestone
C	Ktzl	Thick bedded to massive, white to pinkish orbitolinid-bearing limestone
D	Mur	Red marl, gypsiferous marl, sandstone and conglomerate
	Murc	Red conglomerate and sandstone
	Murm	Light - red to brown marl and gypsiferous marl with sandstone intercalations
E	Olm,s,c	Red and green silty, gypsiferous marl, sandstone and gypsum
F	Pgkc	Light-red coarse grained, polygenic conglomerate with sandstone intercalations
	pCgn	Gneiss, granite gneiss and locally including migmatite
	PlQc	Fluvial conglomerate, Piedmont conglomerate and sandstone.
	pCmt1	Medium-grade, regional metamorphic rocks
	pCmt2	Low - grade, regional metamorphic rocks
G	Qsf	Salt flat
	Qft1	High level piedmont fan and valley terrace deposits
	Qft2	Low level piedmont fan and valley terrace deposits
	Qs,d	Unconsolidated wind-blown sand deposits, including sand dunes
	Qcf	Clay flat
	Qm	Swamp and marsh
H	TRJs	Dark grey shale and sandstone
